# Strategies for monitoring and evaluation of resource-limited national antiretroviral therapy programs: the two-phase design

**DOI:** 10.1186/s12874-015-0027-9

**Published:** 2015-04-07

**Authors:** Sebastien Haneuse, Bethany Hedt-Gauthier, Frank Chimbwandira, Simon Makombe, Lyson Tenthani, Andreas Jahn

**Affiliations:** Department of Biostatistics, Harvard T.H. Chan School of Public Health, Boston, MA USA; Department of Global Health and Social Medicine, Harvard Medical School, Boston, MA USA; Department for HIV and AIDS, Ministry of Health, Lilongwe, Malawi; International Training and Education Center for Health, Lilongwe, Malawi; International Training and Education Center for Health, Department for Global Health, University of Washington, Seattle, USA

**Keywords:** Ecological bias, Aggregated data, Case–control studies, Two-phase sampling, Efficiency

## Abstract

**Background:**

In resource-limited settings, monitoring and evaluation (M&E) of antiretroviral treatment (ART) programs often relies on aggregated facility-level data. Such data are limited, however, because of the potential for ecological bias, although collecting detailed patient-level data is often prohibitively expensive. To resolve this dilemma, we propose the use of the two-phase design. Specifically, when the outcome of interest is binary, the two-phase design provides a framework within which researchers can resolve ecological bias through the collection of patient-level data on a sub-sample of individuals while making use of the routinely collected aggregated data to obtain potentially substantial efficiency gains.

**Methods:**

Between 2005–2007, the Malawian Ministry of Health conducted a one-time cross-sectional survey of 82,887 patients registered at 189 ART clinics. Using these patient data, an aggregated dataset is constructed to mimic the type of data that it routinely available. A hypothetical study of risk factors for patient outcomes at 6 months post-registration is considered. Analyses are conducted based on: (i) complete patient-level data; (ii) aggregated data; (iii) a hypothetical case–control study; (iv) a hypothetical two-phase study stratified on clinic type; and, (v) a hypothetical two-phase study stratified on clinic type and registration year. A simulation study is conducted to compare statistical power to detect an interaction between clinic type and year of registration across the designs.

**Results:**

Analyses and conclusions based solely on aggregated data may suffer from ecological bias. Collecting and analyzing patient data using either a case–control or two-phase design resolves ecological bias to provide valid conclusions. To detect the interaction between clinic type and year of registration, the case–control design would require a prohibitively large sample size. In contrast, a two-phase design that stratifies on clinic and year of registration achieves greater than 85% power with as few as 1,000 patient samples.

**Conclusions:**

Two-phase designs have the potential to augment current M&E efforts in resource-limited settings by providing a framework for the collection and analysis of patient data. The design is cost-efficient in the sense that it often requires far fewer patients to be sampled when compared to standard designs.

## Background

The long-term success of national antiretroviral treatment (ART) programs relies on accurate and timely systems for monitoring and evaluation (M&E). Data from such systems are used for program planning, management of the commodity supply chain, to identify and address emerging implementation or clinical problems, and to facilitate epidemiologic analysis and operations research [1]. With these purposes in mind, M&E systems would ideally record detailed patient-level data on demographic characteristics, medical history, clinical information including virologic and CD4 counts at the time of ART initiation, and outcomes. Toward this ideal, a number of small-scale programs have been successful in establishing infrastructures that routinely collect electronic patient-level data [2-6] and, armed with patient-level data, researchers have been able to address a range of important questions regarding program retention, treatment adherence, drug resistance and mortality [7-13].

Unfortunately, however, implementing comprehensive data collection infrastructures on a national scale in resource-limited settings is prohibitively expensive [14]. In response, the World Health Organization (WHO) public-health approach advocates a simplified strategy for M&E that relies primarily on aggregated, facility-level data [1,3,15]. While these aggregated data represent a critical resource [16-18], they lack the detail and specificity of patient-level data and are therefore limited. In particular, investigations of associations for patient-level outcomes based on aggregated data may suffer from ecological bias [19,20] and, in the worst-case scenario, the ecological fallacy where conclusions based on aggregated data are different than those that would have been drawn had a patient-level analysis been performed [21].

In general, the only reliable approach to overcoming ecological bias is to collect and analyze patient-level data [22]. Fortunately, researchers have at their disposal a broad range of study designs on which to base data collection for a sub-sample of patients. When the outcome is binary, for example, the case–control design is well-known to be efficient relative to random sampling [23]. The case–control design fails, however, to make use of any information other than outcome status. As an alternative we propose strategies for cost-efficient M&E of patient-level outcomes for national ART programs in resource-limited settings based on the two-phase study design [24-27]. As we elaborate upon, two-phase designs provide a framework within which routinely collected aggregated data can be used to identify sub-samples of patients on whom detailed information is collected. To illustrate the design, in terms of both resolving ecological bias and increased statistical power relative to the case–control design, we use data from a cross-sectional survey on the national ART program in Malawi.

## Methods

### The Malawian national ART program

The national ART program in Malawi coordinates care at over 650 clinic sites across the country [28]. Every three months, the Ministry of Health conducts supervision visits to each clinic. During each supervision visit all patients who were newly registered in the previous three months are said to belong to a specific ‘quarterly-clinic cohort’ [28,29]. For all patients in the quarterly-clinic cohort, information recorded on paper-based master cards and stored at the clinic on all patients in each quarterly-clinic cohort is categorized and aggregated. This results in a single record, specific to the entire quarterly-clinic cohort, that includes the number of males/females, the number of adults/children, and the number of patients in different clinical stages. Note, the single record does not include information on the cross-classification of these variables; it does not, example, include separate counts for the number of female adults and male adults. For other quarterly-clinic cohorts at the clinic (i.e. patients registered in previous 3-month periods), aggregated follow-up information such as the total number of retained registrants, the total number of patients who remain adherent to ART, and totals regarding side effects. Finally, ‘cumulative outcomes’ are classified and tallied, giving totals based on the status of patients at their most recent visit before the end of the quarter evaluated. After completing this aggregation process, all quarterly-clinic cohort-specific records are returned to the Ministry of Health, entered into an electronic database and prepared for analysis [30,31].

Between 04/2008 and 05/2009 the Malawian Ministry of Health also conducted a one-time, cross-sectional survey of their national ART program. For each program registrant baseline demographic characteristics (age, gender and WHO stage) were recorded, as well as treatment information (date of ART initiation and current regimen) and information on the clinic (location and clinic type). In addition, the patient’s status at the time of the survey was also recorded. This information was then used to create a binary outcome of ‘status at six months post-registration’: stopped treatment, lost to follow-up and death within 180 days were considered ‘negative’; transferred-out and alive and on-treatment were considered ‘non-negative’.

### Ethics statement

Measures are in place in all ART facilities to ensure patient confidentiality, consent for HIV testing, and counseling and support for those who receive a positive HIV test result. Studies using data collected routinely within the context of monitoring and evaluation, such as ART registers, do not require formal approval by the Malawi National Health Science Research Committee. Prior to data analysis, all individual-level data was completely de-identified. On this basis, the work of this manuscript was determined to be ‘Not Human Subjects Research’ by both the Harvard School of Public Health and the Harvard School of Medicine.

### The potential for ecological bias

As indicated above, the reliance of current systems for M&E on aggregated facility-level data renders analyses open to potential ecological bias. Prior to detailing the use of two-phase designs in the M&E setting, we first motivate the use of the design by illustrating ecological bias in a hypothetical study of the association between clinic type (private vs. public) and outcomes six-month post-registration in the program. Specifically, we constructed and analyzed an (artificially) aggregated dataset using the survey data and compared the results to a “gold-standard” analysis based on the patient-level data. For the latter we fit a logistic regression model to adults (≥16 years) who registered between 2005 and 2007, started ART at registration and had at least six months of follow-up. For simplicity, and to focus the analysis on illustrating ecological bias, we further restricted to patients with non-missing baseline demographic information, yielding an overall sample size of N = 82,877. To provide some adjustment for case-mix differences between patients registered at private and public clinics, we included in the model the following covariates: age, gender, WHO stage at registration and region. Finally, an interaction between clinic type and year of registration was included to investigate whether or not differences between public and private clinics changed over time.

Towards mimicking the current systems in Malawi we first assigned each of the N = 82,877 patients to a quarterly-clinic cohort on the basis of their date and location of registration. For each of the resulting N* = 1,518 quarterly-clinic cohorts we computed a series of aggregated counts/measures including: the total number of registrants, the average age, the number and percent female, the number and percent with WHO stage 3/4 at registration and number and percent with a negative outcome status. To analyze the aggregated quarterly-clinic cohort dataset we fit a logistic regression model with the number of patients with a negative six-month status in the quarterly-clinic cohort as a binomial outcome. The approach to adjustment followed that of the complete patient-level data analysis and included the following group-level covariates: mean age, percent female, an indicator of whether or not the percent WHO stage 3/4 was ≤/> 90%, and region. A main effect for clinic type was included, along with interaction terms with year of registration. To accommodate potential overdispersion, and ensure valid 95% confidence intervals, we used quasi-likelihood for estimation and inference [32].

### Two-phase designs for M&E

In theory, collecting complete patient-level data on a national scale and on a routine basis in Malawi is possible; as mentioned, patient-level data is recorded on paper master cards and stored at each clinic. In practice, however, collecting these data on all registrants of the national ART program is not feasible. As an alternative to attempting to collect patient data on all registrants is to do so on a select sub-sample of patients. In the context of a rare binary outcome, the case–control design is well-known to provide substantial efficiency gains relative to simple random sampling [23]. In Malawi, a case–control study could easily be implemented by stratifying the registrant population (i.e. the N = 82,887 patients) on the known number of cases and non-cases (N_1_ = 16,141 and N_0_ = 66,746; see Table 1), selecting a random sub-sample from each outcome-specific strata and transferring data for the selected patients from their master cards into an electronic format for analysis.

**Table 1:** **Patient outcomes at six months, by patient and clinic characteristics as well as by year of registration**

	**Status at six months**		**Rate, %**
	**Non-negative** ^**a**^	**Negative** ^**b**^	
**Overall**	66,746	16,141	19.5
**Age, years**			
16-25	7,116	2,130	23.0
26-35	26,460	6,575	19.9
36-45	21,078	4,756	18.4
46-55	8,835	1,888	17.6
56-65	2,785	656	19.1
>65	472	136	22.4
**Gender**			
Male	25,150	7,172	22.2
Female	41,596	8,969	17.7
**WHO stage**			
1/2	4,418	314	6.6
3/4	62,328	15,827	20.3
**Region**			
Central/North	27,662	7,269	20.8
South	39,084	8,872	18.5
**Registration year**			
2005	12,238	3,514	22.3
2006	23,893	6,181	20.6
2007	30,615	6,446	17.4
**Clinic type**			
Public	64,651	15,839	19.7
Private	2,095	302	12.6

^a^Non-negative = transferred-out or alive and on-treatment.

^b^Negative = stopping treatment, lost to follow-up or death.

While the patient-level data obtained via the case–control design can be used to resolve ecological bias, it makes no use of the routinely collected aggregated quarterly-clinic cohort data. Two-phase designs provide a framework for using these data [27]. In the Malawian context, phase I would correspond to a stratification of the entire population on the basis of outcome status (as in a case–control design) and the known aggregated quarterly-clinic cohort data. Table 2 provides six possible phase I stratifications for the N = 82,877 patients. Design #1 exploits the fact that whether a clinic is private or public is common to all patients in the quarterly-clinic cohort. Consequently, it is possible to cross-classify all patients (across all N* = 1,518) by outcome status and type of clinic. Similarly, since each quarterly-clinic cohort is specific to 2005, 2006 or 2007 it if possible to further cross-classify the counts by year of registration as in Design #2. In Design #3, the cross-classification exploits the fact that all patients in a quarterly-clinic cohort “share” the common prevalence of WHO stage 1 or 2, even though the values vary within the quarterly-clinic cohort. Similarly in Designs #4 and Designs #5 for the “shared” average age and percent female in the quarterly-clinic cohort. Finally, Design #6 further illustrates the potential for combining two group-level covariates to more finely stratify the phase I sample.

**Table 2:** **Six possible phase I stratification schemes that use readily-available group-level information collected by the current M&E systems in Malawi**

**Design #1**	Private clinic					
	No	Yes				
Non-negative status	64,651	2,095				
Negative status	15,839	302				
**Design #2**	Year of registration/Private clinic					
	2005/No	2005/Yes	2006/No	2006/Yes	2007/No	2007/Yes
Non-negative status	11,991	247	22,887	1,006	29,773	842
Negative status	3,492	22	6,104	167	6,333	113
**Design #3**	Percent WHO stage 1 or 2					
	≤5%	>5%				
Non-negative status	50,570	16,176				
Negative status	13,191	2,950				
**Design #4**	Average age, years					
	≤35	36-40	>40			
Non-negative status	12,954	51,959	1,833			
Negative status	3,570	12,239	332			
**Design #5**	Percent female					
	0%	1-40%	41-50%	51-60%	60-99%	100%
Non-negative status	189	3,360	4,766	19,255	38,949	227
Negative status	20	630	1,144	5,248	9,081	18
**Design #6**	Percent WHO stage 1 or 2/Private clinic					
	≤5%/No	>5%/No	≤5%/Yes	>5%/Yes		
Non-negative status	49,160	15,491	1,410	685		
Negative status	12,796	2,863	215	87		

Focusing on Designs #1 and #2 for the remainder of this paper, given a phase I stratification scheme, sub-samples of patients are chosen from each of the phase I strata and, as in a case–control design, detailed patient data is retrospectively ascertained. These data are collectively referred to as the phase II data. In practice, the number of patients sampled at phase II is typically fixed and one must decide how to allocate those resources across the phase I strata. One straightforward strategy is to adopt a balanced design that allocates them equally. For Design #1, given resources to collect a sub-sample of n = 5,000, a balanced design would collect 1,250 patients from each phase I strata. Since only 302 patients were registered at private clinics and had a negative outcome, all of these patients would be sampled; the remaining 2,198 ‘cases’ would then be sampled from public clinics. Similarly, for a fixed phase II sample size of n = 5000, balanced sampling would draw 416 patients from each of the 12 phase I strata in Design #2. As in Design #1, some strata do not contain sufficient patients and the remainder could be drawn from the other (outcome-specific) strata.

Given data from a two-phase design, analysts can use any of a number of different approaches to estimation and inference for an underlying logistic regression model, including weighted likelihood, pseudo-likelihood and maximum likelihood [33,34]. Each of these approaches have been implemented and are currently available in the osDesign package for R [35].

### A simulation to investigate statistical power

To further illustrate the potential and benefit of the two-phase design, we performed a series of simulations to investigate statistical power. Specifically, we generated 1,000 simulated datasets each of size N = 82,887 and with the same covariate distribution as the survey data. Outcomes were generated as Bernoulli random draws with a patients’ probability determined by the “gold-standard” logistic regression analysis of the patient-level data in the survey.

For each dataset, and for a range of sub-sample sample sizes, we simulated a case–control study and the two two-phase designs described above. Note, since the outcomes are simulated, they vary from dataset to dataset; as such, the observed actual phase I stratification varied from dataset to dataset. For each dataset, we then estimated the regression parameters from the underlying logistic regression model using maximum likelihood and evaluated whether or not the interaction terms were statistically different from zero (based on a Wald test with 2 degrees of freedom). Statistical power was evaluated as the proportion of instances in which the null hypothesis of no interaction was rejected.

## Results

Table 3 provides a summary of the data observed in the survey; the left-hand side summaries patient-level characteristics of the N = 82,877 patients; the right-hand summaries the group-level data for the N* = 1,518 quarterly-clinic cohorts. From the left-hand side, we see that 9,246 of the N = 82,887 patients (11.2%) were aged 16–25 years, 4,049 (4.9%) were older than 55 years, 50,565 (61%) were female and the vast majority (94.3%) presented at WHO stage 3 or 4. From the right-hand size, 62 (4.1%) of the N* = 1,518 cohorts had an average age of ≤30 years, while 23 (1.5%) had an average age >50 years. One hundred and fourteen cohorts (7.5%) were all male and 135 (8.9%) were all female. For the majority of cohorts (1,263; 83.2%) the prevalence of a WHO stage of 3/4 at registration was ≥90%. Finally, 301 cohorts (19.8%) were at private clinics. Note, from the left-hand side of Table 3 these cohorts accounted for 2,397 (2.9%) of the patients.

**Table 3:** Characteristics of N = 82,877 patients and the corresponding N* = 1,518 quarterly-clinic cohorts, from a cross-sectional survey conducted in Malawi between 04/2008-05/2009

**Patients**			**Quarterly-clinic cohorts**		
	**N**	**%**		N*	**%**
**Total**	82,887		**Total**	1,518	
**Age, years**			**Average age, years**		
16-25	9,246	11.2	≤30	62	4.1
26-35	33,035	39.9	31-35	344	22.7
36-45	25,834	31.2	36-40	921	60.7
46-55	10,723	12.9	41-45	137	9.0
56-65	3,441	4.2	46-50	31	2.0
>65	608	0.7	>50	23	1.5
**Gender**			**Percent female**		
Male	32,322	39.0	0%	114	7.5
Female	50,565	61.0	1-40%	123	8.1
			41-50%	201	13.2
			51-60%	339	22.3
			60-99%	606	39.9
			100%	135	8.9
**WHO stage**			**Percent WHO stage 3/4**		
1/2	4,732	5.7	≤90%	255	16.8
3/4	78,155	94.3	>90%	1,263	83.2
**Region**			**Region**		
Central/North	34,931	42.1	Central/North	720	47.4
South	47,956	57.9	South	798	52.6
**Year of registration**			**Year of registration**		
2005	15,752	19.0	2005	334	22.0
2006	30,074	36.3	2006	560	36.9
2007	37,061	44.7	2007	624	41.1
**Clinic type**			**Clinic type**		
Public	80,490	97.1	Public	1,217	80.2
Private	2,397	2.9	Private	301	19.8

The overall six-month negative outcome rate was 19.5% (Table 1). The rate was highest among younger and older patients, with patients aged 46–55 years experiencing the lowest rate (17.6%). Furthermore, the rate was lower among females (17.7% versus 22.2% for males), among patients with WHO stage 1/2 (6.6% versus 20.3% among patients with WHO stage 3/4) and among patients registered at private clinics (12.6% versus 19.7% among patients at public clinics).

From the “gold-standard” analysis, presented in the first column of Table 4, we see that patients at private clinics have substantially lower adjusted odds of a negative six-month outcome. In particular, the odds of a negative outcome for a patient registered at a private clinic in 2005 are estimated to be 69% lower than the odds of a patient registered at a public clinic in 2005 (OR 0.31; 95% CI 0.20, 0.48). Furthermore, the interaction terms are statistically significant (overall p-value < 0.01). Combining the main effect and interaction terms, the year-specific private/clinic ORs in 2006 and 2007 are 0.67 (95% CI 0.56, 0.79) and 0.63 (0.52, 0.77), respectively, indicating that while the gap between private and public clinics diminished from 2005 to 2006, it did not diminish completely and remained constant through 2007.

**Table 4:** **Estimated odds ratios (OR) and 95% confidence intervals (CI) from logistic regression models for a negative outcome status at six months, based on five scenarios for patient and aggregated data availability**

	**Complete patient data (N = 82,887)**		**Aggregated quarterly-clinic data (N = 1,518)**		**Case–control study (N = 5,000)**		**Two-phase study** ^**d**^			
							**Design #1 (N = 5,000)**		**Design #2 (N = 5,000)**	
	**OR**	**95% CI**	**OR**	**95% CI**	**OR**	**95% CI**	**OR**	**95% CI**	**OR**	**95% CI**
**Age** ^**a**^										
Linear	0.96	(0.94, 0.98)	0.35	(0.19, 0.66)	0.94	(0.25, 0.52)	0.94	(0.87, 1.01)	0.88	(0.80, 0.95)
Quadratic	1.06	(1.05, 1.07)	0.77	(0.54, 1.10)	1.05	(0.88, 1.00)	1.04	(0.99, 1.08)	1.03	(0.98, 1.08)
**Gender**										
Male	REF		REF		REF		REF		REF	
Female^b^	0.72	(0.69, 0.74)	0.87	(0.82, 0.93)	0.75	(0.66, 0.84)	0.76	(0.67, 0.87)	0.71	(0.62, 0.82)
**WHO stage**										
1/2	REF		REF		REF		REF		REF	
3/4^c^	3.33	(2.97, 3.74)	1.57	(1.44, 1.72)	3.69	(2.62, 5.20)	3.31	(2.41, 4.55)	4.31	(2.87, 6.46)
**Region**										
Central/North	REF		REF		REF		REF		REF	
South	0.92	(0.88, 0.95)	0.88	(0.82, 0.93)	0.93	(0.83, 1.05)	0.91	(0.80, 1.04)	1.02	(0.88, 1.17)
**Year of registration**										
2005	REF		REF		REF		REF		REF	
2006	0.91	(0.87, 0.95)	1.00	(0.94, 1.07)	0.97	(0.83, 1.05)	0.83	(0.68, 1.01)	0.88	(0.83, 0.93)
2007	0.76	(0.73, 0.80)	0.91	(0.85, 0.97)	0.74	(0.63, 0.86)	0.75	(0.62, 0.91)	0.75	(0.71, 0.80)
**Clinic type**										
Public	REF		REF		REF		REF		REF	
Private	0.31	(0.20, 0.48)	1.08	(0.99, 1.19)	0.39	(0.13, 1.14)	0.31	(0.19, 0.50)	0.31	(0.20, 0.47)
**Clinic/year interaction**										
Private/2006	2.16	(1.35, 3.46)	2.03	(0.95, 4.35)	2.20	(0.67, 7.28)	2.18	(1.30, 3.67)	2.20	(1.37, 3.53)
Private/2007	2.05	(1.26, 3.32)	1.98	(0.91, 4.32)	2.36	(0.69, 8.05)	2.07	(1.21, 3.52)	1.98	(1.22, 3.21)

^a^Quarterly-clinic cohort data model uses average age (in years).

^b^OR for the quarterly-clinic cohort data model corresponds to a contrast of 20% in the percent female.

^c^Covariate in the quarterly-clinic cohort data model is a binary indicator of whether or not the the percent WHO stage 3/4 is ≤/> 90%.

^d^See Table 2 for details; Design #1 stratifies on clinic type; Design #2 stratifies on clinic type and year of registration.

**Figure 1:**
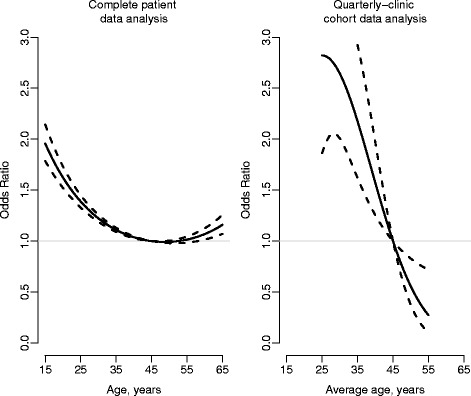
Results on the association between age and negative outcome status based on the complete patient data (N = 82,877 patient records) and the quarterly-clinic cohort data (N* = 1,518 records). Shown are odds ratio estimates and 95% confidence intervals; the referent age level for the odds ratio associations is 45 years.

The second column of Table 4 provides results based on the group-level analysis of the quarterly-clinic cohort data. Comparing with the first column, we see discrepant results between the patient- and group-level analyses for the effects of age, gender and WHO stage. For gender and WHO stage, the point estimates based on the aggregated data analysis are substantially attenuated, although remain statistically significant. Analyses based on patient-level data indicate a statistically significant U-shaped relationship between age and six-month outcomes (see Figure 1). Analyses based on group-level data fail to identify a statistically significant quadratic term and erroneously suggest a linear effect for age. This is a classic manifestation of ecological bias.

The third column of Table 4 provides results based on a single case–control draw of n = 5,000 patients from the N = 82,877 available in the survey. Overall the results based on the case–control data and the gold-standard analyses are consistent with each other, despite the former only requiring detailed data on a fraction (5,000 of 82,877; 6%) of the patient records. The fourth and fifth columns of Table 4 provide results based on a single phase II draw under Designs #1 and #2. As with the case–control study, both sets of results are consistent with the gold-standard complete patient data analyses. One crucial difference, however, is that the confidence intervals for the clinic effect are much tighter under the two two-phase designs; compare (0.13, 1.14) to (0.19, 0.50) and (0.20, 0.47). Indeed, the results/conclusions for clinic type based on the two-phase designs are almost equivalent to those based on the gold standard even though the former uses a fraction (again, 5,000/82,877; 6%) of the patient data. Similarly, compared to the case–control design, the estimates/conclusions for the two interaction terms are substantially improved under either of the two-phase designs.

**Figure 2:**
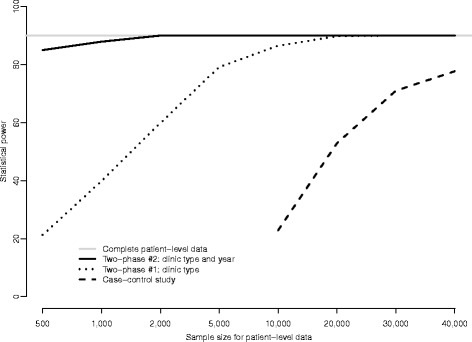
**Estimated post-hoc power to detect an interaction between clinic type and year of registration under (i) a gold-standard complete data design with patient-level data on all N = 82,877 patients; (ii) a case–control design; (iii) a two-phase design, stratifying on clinic type; and, a two-phase design, stratifying on clinic type and year of registration.**

Figure 2 provides the results from the simulation study. The grey line indicates that analyses based on the complete data (i.e. N = 82,877) had approximately 90% power to detect the clinic/year interaction. From the Figure we see that a case–control design with n = 10,000 patients would only have approximately 23% power. Increasing the case–control sample size to n = 20,000 only increases power to 53%; at n = 40,000, power is approximately 80%. In comparison, one would only need n = 5,000 phase II samples under two-phase Design #1 to have approximately 80% power. Under Design #2, a phase II sample size as low as n = 500 would provide more than 85% power to detect the clinic/year interaction. Furthermore, when the phase II sample size is n = 2,000, Design #2 has equivalent statistical power to a study in which patient-level data was collected on all N = 82,877 patients.

## Discussion

Given significant financial constraints, effective and comprehensive monitoring remains a critical challenge for many national ART programs. There is therefore a pressing need for innovative strategies that are robust to ecological bias and that permit M&E of patient-level outcomes and their associations with risk factors. The two-phase study design is one such strategy, providing a cost-efficient approach to combining and making best use of two sources of information: the existing aggregated group-level counts and the sub-samples of patient-level data. Using relatively recent advances in statistical methodology, the designs are flexible and can often permit the investigation of patient-level outcomes/associations with detailed information on only a fraction of patient registrants. This core feature provides important flexibility for resource-limited settings where minimizing costs is a major concern.

The example used to illustrate the two-phase design considered a very specific question: the relationship between clinic type (public/private) and outcomes, and whether or not the relationship varied over time. In general, stratification improves power for detecting effects association with the stratification variables. This manifested in our example through the enormous improvements in power when the phase I stratification was based on clinic type and year of registration. There is, however, a trade-off, in that statistical power can be reduced for effects associated variables that are not involved in the stratification. This phenomenon arises in the analyses of Table 4 where standard error estimates for the age, WHO stage and region coefficients are all approximately 20% bigger under the two-phase designs than the case–control design. This highlights the importance of careful study design when choosing the phase I stratification scheme and gearing it to the goals of the study. It also highlights the important distinction between one-time studies of some specific question, such as the one considered in this paper, and more general on-going M&E efforts. For the latter, in which data from sub-samples of patients may be routinely collected the choice of phase I stratification will need to be tailored to more general sets of goals. How this is done remains an open question and represents an important avenue for future research.

The overarching goal of this paper is to emphasize the value added to aggregate program data with the use of a two-phase design. Beyond the two-phase design, the survey sampling literature provides a broad range of strategies for collecting individual-level data [36,37]. One could, for example, perform clustered sampling in which a random sample of clinics is chosen and then a random sample of patients within each clinic is identified. Such an approach is useful from a logistical perspective since individual-level data need only be collected from a limited number of clinics. One benefit of the two-phase design in the Malawian context, however, is the flexible, explicit use of the aggregated data via the design (i.e. phase I stratification) and the recently developed efficient analyses techniques [25,26].

The data used for this illustrative purpose had several limitations in terms of missing data and limited data fields, and may suffer from additional data quality issues that are common in the field (e.g. misclassification and measurement error). The results themselves are illustrative only and are not intended to be generalizable to either the Malawian national ART program or beyond. Further, while there are discrepancies in results based on the patient-level data compared to the aggregated datasets, the intention of this paper is not to undermine the critical role of the aggregated program data. Certainly there is precedence in the use of this aggregated data to monitor patient outcomes, forecast program need, and make statements about the utility of national treatment programs. Crucially, it is when aggregated data are used to to make statements about more complex relationships between exposures and outcomes that bias and the ecological fallacy can arise. Whether or not the underlying quality of the aggregated data impacts the efficiency gains of a two-phase study is an open design question and one we are actively pursuing.

Throughout, we have sought to emphasize the practical utility of two-phase designs in making efficient use of information that already exists (i.e. the information already collected by the Malawian national ART program). The statistical literature laying out the theoretical foundation for these designs is rich, with much of the development in the last 20 years [25-27,34,38], In the context of this paper, as pointed out by a reviewer, program registrants are clustered within clinics and, as such, a complete data analysis would require acknowledging this phenomenon to ensure valid inference. Interestingly, the literature on two-phase designs focuses exclusively on settings where individual study units are independent; that is, the context where study units are cluster-correlated has not been considered for two-phase designs. Indeed, to our knowledge, no statistical methods have been published for data arising from a standard case–control design when the underlying patient population exhibits clustering. As such, we have not considered the potential effects of clustering. With respect to the key messages of this paper, however, such clustering does not impact the notion that individual-level data can be used to alleviate ecological bias and it is unlikely to impact the relative differences in statistical efficiency/power between the case–control and two-phase design. Towards the latter, we are actively developing methods for cluster-correlated case–control and two-phase designs, as well as case–control designs where a fixed number of cases and controls are selected from each clinic [39].

Finally, while this work is motivated by challenges posed to the Malawian national ART program, the approach will be useful in a wide array of resource-limited settings both, at the national and local scale. In particular, the availability of this strategy could help inform decisions on monitoring efforts faced by (i) other programs that currently use aggregated data for monitoring, (ii) programs that collect patient-level data but where certain data elements are either missing or subject to measurement error; and, (iii) programs that currently collect comprehensive patient-level data but are interested in strategies to reducing costs.

## Conclusions

Currently, ART programs in resource-limited settings rely on aggregated facility-level data to perform M&E and are therefore subject to potential ecological bias. Two-phase designs provide a flexible framework for judiciously collecting sub-samples of patient-level data. Specifically, by making use of existing data collection efforts to form efficient sampling frames, the two-phase design permits the resolution of ecological bias, giving researchers the ability to address a broad range of patient-level questions. Furthermore, the design is cost-efficient in the sense that, when compared to standard designs such as the case–control study, far fewer patients need to be sampled to achieve a desired level of statistical efficiency and power.

## Abbreviations

ART: Anti-retroviral treatment
M&E: Monitoring and evaluation
WHO: World Health Organization
OR: Odds ratio
CI: Confidence interval
